# Real-time diagnostic analysis of MinION™-based metagenomic sequencing in clinical microbiology evaluation: a case report

**DOI:** 10.1186/s40981-019-0244-z

**Published:** 2019-03-19

**Authors:** Hiromasa Tanaka, Yoshiyuki Matsuo, So Nakagawa, Kenichiro Nishi, Akihisa Okamoto, Shinichi Kai, Teppei Iwai, Yoshiteru Tabata, Takeshi Tajima, Yuji Komatsu, Motohiko Satoh, Kirill Kryukov, Tadashi Imanishi, Kiichi Hirota

**Affiliations:** 1 Department of Human Stress Response Science, Institute of Biomedical Science, Kansai Medical University, Hirakata, Japan; 2 Department of Molecular Life Science, Tokai University School of Medicine, Isehara, Japan; 3 Department of Anesthesiology, Osaka Red Cross Hospital, Osaka, Japan; 4 Department of Anesthesia, Kyoto University Hospital, Kyoto, Japan; 5 Meisei Hospital, Osaka, Japan

To the editor,

Rapid identification of causative pathogenic bacteria is crucial for the treatment of patients with infectious diseases, including pneumonia [1]. For this purpose, molecular techniques of genetic diagnosis of infectious diseases have been developed and applied to rapid diagnostic procedures [2, 3]. In this report, we conducted 16S ribosomal RNA (rRNA) gene amplicon sequencing and metagenomic analysis of DNA extracted from airway secretion of a patient suffering from acute respiratory distress syndrome (ARDS) as a consequence of pneumonia by using the portable DNA sequencer MinION™ [4, 5] (see Additional file 1).

## Case presentation

**Fig. 1:**
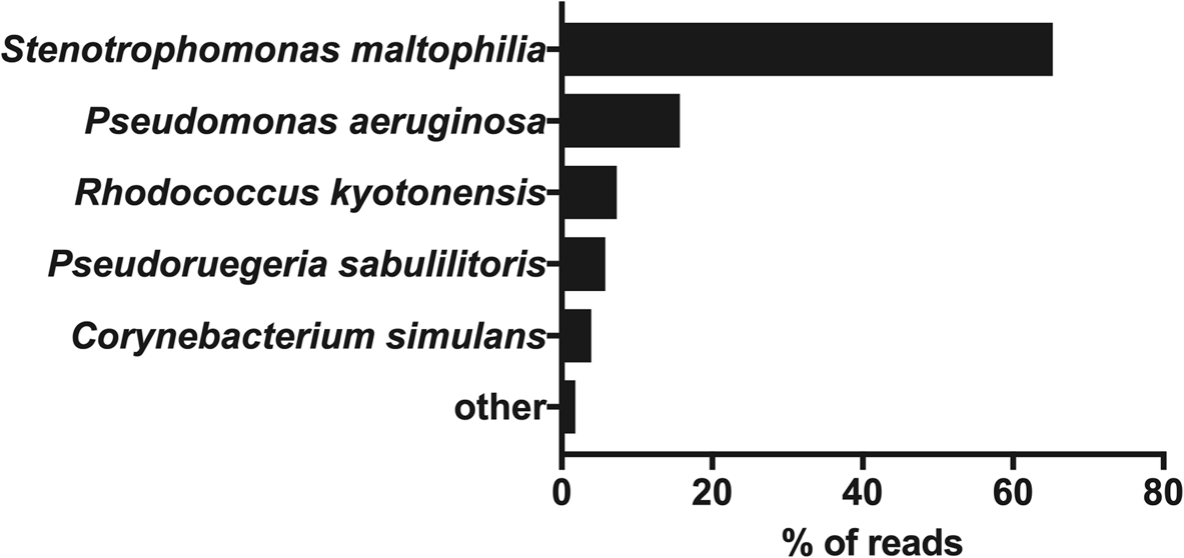
Taxonomic assignment of the 16S rRNA genes in sputum samples. The samples were subjected to 16S rRNA amplicon sequencing using MinION™ (Oxford Nanopore Technologies, Oxford, UK), and the percentage of reads (with the abundance over 2%) belonging to the identified bacterial species is shown. Sequencing for 5 min generated 470,231 reads. A total of 41,136 reads were aligned with one of the bacterial 16S rRNA gene sequences, and 65.3% reads were aligned with *Stenotrophomonas maltophilia* and 15.7% reads were aligned with *Pseudomonas aeruginosa*

A 68-year-old man with a past history of schizophrenia underwent laparoscopic right hemicolectomy due to carcinoma. At postoperative day 4, he was diagnosed with aspiration pneumonia as a consequence of vomiting due to intestinal obstruction and septic shock. He was transferred to the intensive care unit for further observation and treatment (see Additional file 2). At day 46, we performed microbial laboratory evaluation based on both bacterial culture and 16S rRNA gene amplicon sequencing analysis following the pipeline established by us previously [4, 5] (see Additional file 3). MinION™-based sequencing analysis just 2 h after sample collection revealed the presence of *Stenotrophomonas maltophilia*, *Pseudomonas aeruginosa*, *Rhodococcus kyotonensis*, *Pseudoruegeria sabulilitoris*, *Corynebacterium simulans*, and other microorganisms (see Additional file 4) (Fig. 1). Culture-based testing reported at 2 days thereafter detected *Stenotrophomonas maltophilia* and *Pseudomonas aeruginosa*.

## Discussion

We demonstrated the feasibility of the tentative point-of-care diagnosis by 16S rRNA amplicon sequencing via the MinION™ sequencer and subsequent confirmation of the results by standard culture methods. To facilitate the process of sample preparation, we amplified 16S rRNA genes directly from the specimen without DNA purification and after mechanical disruption treatment by bead-beating [6]. With the MinION™ sequencer, generated reads can be analyzed in real time, which makes this approach all the more promising [4]. In the present case, the identification process took only 2 h, including PCR amplification of 16S rRNA genes, sequencing on the MinION™, and bioinformatics analyses. The sequencing-based diagnostic approach is more sensitive than conventional culture-based tests. This feature can be useful for identifying unculturable bacteria or detecting bacteria in specimens after exposure to antimicrobial treatments.

Another advantage of the MinION™ sequencer is the long length of the read [7]. Conventional next-generation sequencing (NGS) generates relatively short reads with limited sequence information that do not allow distinguishing clearly between different bacterial species. The nanopore sequencing enables us to cover the entire region of the 16S rRNA gene, so detection on the species level has become possible (see Additional file 5) [5].

Further studies with more cases are needed to establish reliable diagnostic criteria based on the relative abundance of respiratory pathogen reads compared to those of the commensal bacteria. Clinical sequencing aided by this kind of analysis pipeline may open a way to precision medicine in the field of critical care medicine.

## Additional files

Additional file 1: Nanopore-based MinION™ sequencer and the analysis system. (DOCX 131 kb)

Additional file 2: Computed axial tomography in the intensive care unit. (DOCX 597 kb)

Additional file 3: The pipeline of the analysis. The file is deposited at https://doi.org/10.6084/m9.figshare.7380068. (DOCX 14 kb)

Additional file 4: List of identified bacteria. The file is deposited at https://doi.org/10.6084/m9.figshare.7380074. (XLSX 10 kb)

Additional file 5: Advantages of MinION™-based metagenomic sequencing. (DOCX 97 kb)

## Abbreviations

ARDS: Acute respiratory distress syndrome
NGS: Next-generation sequencing
rRNA: Ribosomal RNA
