# Supplementary figures and images for: Real-time diagnostic analysis of MinION™-based metagenomic sequencing in clinical microbiology evaluation: a case report
Source: JA Clin Rep. 2019 Mar 19;5:24. doi: 10.1186/s40981-019-0244-z (PMC6967274; doi:10.1186/s40981-019-0244-z)

Additional file 1

Nanopore-based MinION™ sequencer (A) and the anslysis system(B)


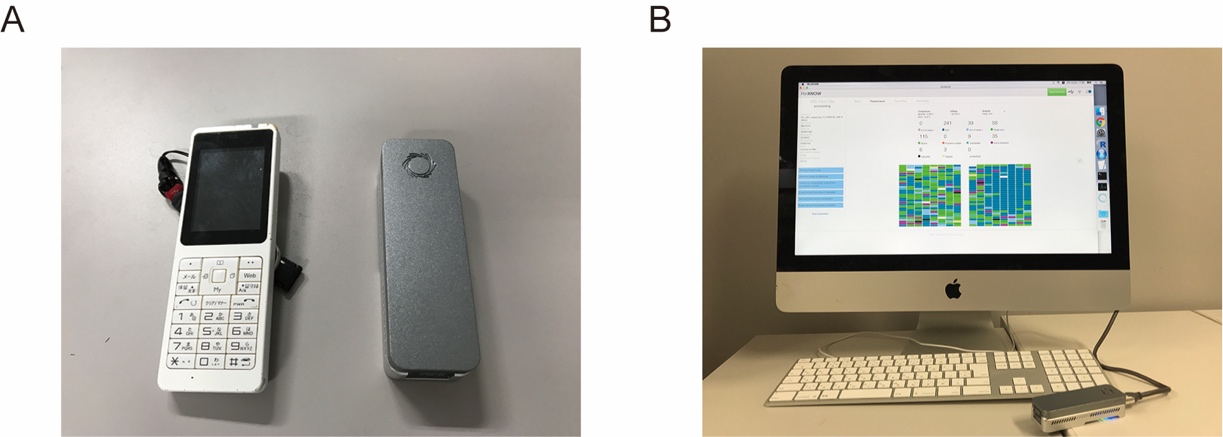

Supplement: Supplementary file 1 — Nanopore-based MinION™ sequencer and the analysis system. (DOCX 131 kb) [file 40981_2019_244_MOESM1_ESM.docx]
