# Supplementary material for: Real-time diagnostic analysis of MinION™-based metagenomic sequencing in clinical microbiology evaluation: a case report
Source: JA Clin Rep. 2019 Mar 19;5:24. doi: 10.1186/s40981-019-0244-z (PMC6967274; doi:10.1186/s40981-019-0244-z)
Supplement: Supplementary file 2 — Computed axial tomography in the intensive care unit. (DOCX 597 kb) [file 40981_2019_244_MOESM2_ESM.docx]

Additional file 2

Computed axial tomography in the intensive care unit.

(A and B) Computed axial tomography scans in which mixed basal pulmonary infiltrates and alveolar collapse were observed. Images were taken at the time of admission (day 1) (A) and day 46 (B).

On the day of admission to ICU, SpO_2_ was 80% under mask with 6L O_2_ in the ward. P/F ration immediate after inbubation was 226 mmHg, SOFA score was 11 and APACHEII score was 38.
