# Supplementary material for: Real-time diagnostic analysis of MinION™-based metagenomic sequencing in clinical microbiology evaluation: a case report
Source: JA Clin Rep. 2019 Mar 19;5:24. doi: 10.1186/s40981-019-0244-z (PMC6967274; doi:10.1186/s40981-019-0244-z)
Supplement: Supplementary file 3 — The pipeline of the analysis. The file is deposited at https://doi.org/10.6084/m9.figshare.7380068. (DOCX 14 kb) [file 40981_2019_244_MOESM3_ESM.docx]

**The pipeline of the analysis**

**Sample preparation and PCR amplification of 16S rRNA genes**

A sputum sample (approximately 0.1 g) was mixed with 500 µl of water, vortexed and centrifuged at 50 × g for 5 min. The supernatant was re-centrifuged at 18,000 × g for 5 min to collect bacteria, and the resulting pellets were suspended in 100 µl of water. For mechanical cell lysis, zirconia beads (EZ-Beads^TM^; Promega, Madison, WI) were added to the suspension and the sample was subjected to four rounds of bead-beating for 30 s with a vortex mixer. The lysate was cleared by centrifugation at 18,000 × g for 1 min, and the supernatant was used as a template for amplifying bacterial 16S rRNA genes. PCR amplification was performed using the 16S Barcoding Kit (SQK-RAB204; Oxford Nanopore Technologies, Oxford, UK) and KAPA2G^TM^ Robust HotStart ReadyMix PCR Kit (Kapa Biosystems, Wilmington, MA). Amplification conditions were as follows, initial denaturation at 95 °C for 3 min, 25 cycles of 95 °C for 15 s, 55 °C for 15 s, 72 °C for 30 s, followed by a final extension at 72 °C for 1 min.

**Library preparation and sequencing of 16S rRNA amplicons**

PCR products were cleaned using AMPure XP (Beckman Coulter, Indianapolis, IN) and eluted in 10 mM Tris-HCl pH 8.0 with 50 mM NaCl. Library preparation was conducted with the 16S Barcoding Kit according to manufacturer’s instructions, and the sample was sequenced on the MinION^TM^ Mk1B sequencer (Oxford Nanopore Technologies) using R9.4 flow cells (FLO-MIN106; Oxford Nanopore Technologies). MinKNOW ver. 1.11.5 (Oxford Nanopore Technologies) was used for data acquisition. Nanopore sequencing and data analysis were performed on Apple iMac (macOS High Sierra 10.13.4; 8 GB memory; 2.9 GHz Intel Core i5 processor).

**Data analysis**

Raw sequencing data in FAST5 format were converted into FASTQ files using Albacore software ver. 2.2.4 (Oxford Nanopore Technologies). The bacterial sequence reads were extracted and taxonomy assignment was performed using minimap2 to search against bacterial genome sequences stored in the GenomeSync database (http://genomesync.org) as described previously.
