# Supplementary material for: Real-time diagnostic analysis of MinION™-based metagenomic sequencing in clinical microbiology evaluation: a case report
Source: JA Clin Rep. 2019 Mar 19;5:24. doi: 10.1186/s40981-019-0244-z (PMC6967274; doi:10.1186/s40981-019-0244-z)

Additonal File 5

Advantages of MinION™-based metagenomic sequencing

Direct amplification of 16S rRNA genes combined with MinION™ sequencing provides an attractive option for rapid detection of bacteria compared to conventional culture-based evaluation. Our simple workflow for rapid bacterial identification may reduce the time from sample collection to result and provides a reliable method applicable to clinical settings.

% of Reads: Proportion of reads assigned to each taxonomic group of bacteria

GSTK: Genome Search Toolkit with GenomeSync database Advantages of MinION™-based metagenomic sequencing


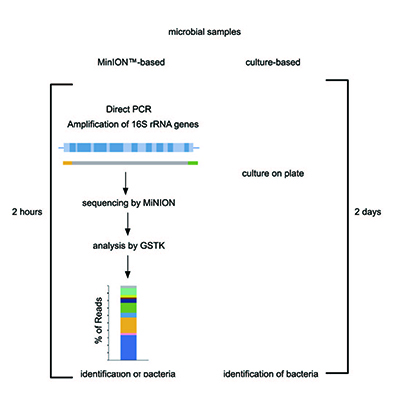

Supplement: Supplementary file 5 — Advantages of MinION™-based metagenomic sequencing. (DOCX 97 kb) [file 40981_2019_244_MOESM5_ESM.docx]
